# *Posidonia oceanica* (L.) Delile as a Marine Anti-Inflammatory Modulator of Keratinocyte Inflammatory Responses Relevant to Psoriasis

**DOI:** 10.3390/md24020085

**Published:** 2026-02-19

**Authors:** Marzia Vasarri, Donatella Degl’Innocenti, Matteo Lulli, Nicola Schiavone, Alice Verdelli, Marzia Caproni, Emiliano Antiga, Emanuela Barletta

**Affiliations:** 1 Department of Experimental and Clinical Biomedical Sciences “Mario Serio”, Section of Biochemistry, University of Florence, Viale G.B. Morgagni 50, 50134 Firenze, Italy; marzia.vasarri@unifi.it (M.V.); donatella.deglinnocenti@unifi.it (D.D.); 2 Interuniversity Center of Marine Biology and Applied Ecology “G.Bacci” (CIMB), Viale N. Sauro 4, 57128 Livorno, Italy; 3 Department of Experimental and Clinical Biomedical Sciences “Mario Serio”, Section of Experimental Pathology and Oncology, University of Florence, Viale G.B. Morgagni 50, 50134 Firenze, Italy; matteo.lulli@unifi.it (M.L.); nicola.schiavone@unifi.it (N.S.); 4 Rare Dermatological Diseases SOS, Central Tuscany Local Health Authority, Viale Michelangiolo 41, 50125 Firenze, Italy; alice.verdelli@uslcentro.toscana.it; 5 Department of Health Sciences, Section of Dermatology, University of Florence, Viale Michelangiolo 41, 50125 Firenze, Italy; marzia.caproni@unifi.it (M.C.); emiliano.antiga@unifi.it (E.A.)

**Keywords:** *Posidonia oceanica*, keratinocytes, skin inflammation, pro-inflammatory cytokines, antioxidants, marine natural products, complementary medicine, lipopolysaccharide

## Abstract

Skin inflammation is characterized by oxidative stress, excessive keratinocyte activation, and the overproduction of pro-inflammatory cytokines. In a previous study, we demonstrated that the hydroalcoholic extract from *Posidonia oceanica* leaves (POE) mitigates psoriasis-like skin inflammation in a mouse model. In the present study, we investigated the cellular mechanisms underlying these effects in human HaCaT keratinocytes. Non-cytotoxic lipopolysaccharide (LPS) stimulation reproduced key inflammatory features, including impaired cell proliferation, increased production of ROS and NO, and the upregulation of IL-1β, IL-6, TNF-α and CXCL8/IL-8. Co-treatment with POE significantly attenuated these alterations by restoring cell proliferation, suppressing oxidative stress, particularly NOS2/NO, and normalizing both cytokine expression and release. POE alone did not affect cell viability or inflammatory markers, confirming its favorable safety profile. However, POE alone induced a mild pro-apoptotic response, which may contribute to overcoming the apoptosis resistance typically observed in psoriatic keratinocytes. Overall, these findings demonstrate that POE exerts antioxidant and anti-inflammatory effects in activated keratinocytes and support its potential as a marine-derived candidate for complementary strategies in the management of psoriasis-associated inflammatory skin disorders.

## 1. Introduction

Skin inflammation is a hallmark of numerous dermatological disorders, including atopic dermatitis, contact dermatitis, psoriasis, and other inflammatory skin conditions. These diseases are characterized by oxidative stress, dysregulated keratinocyte activity, and excessive production of pro-inflammatory cytokines, collectively contributing to epidermal barrier dysfunction and chronic inflammation. Once considered inert structural cells, epidermal keratinocytes are now recognized as key initiators and amplifiers of skin inflammation. They actively contribute to the inflammatory milieu by secreting cytokines such as IL-1, IL-6, TNF-α, and the chemokine CXCL8/IL-8 [1,2].

In psoriasis, the inflammatory response is largely mediated by lymphocytes, particularly Th17 cells, which are activated by exogenous and endogenous stimuli and secrete cytokines including IL-17, IFN-γ, and IL-22. These cytokines drive keratinocyte hyperproliferation, promote anti-apoptotic signaling, and induce aberrant differentiation, leading to psoriatic plaque formation [3]. Increasing evidence indicates that innate immune activation precedes adaptive responses in psoriasis. Stimulation of Toll-like receptors (TLRs) by damage-associated molecular patterns (DAMPs) or pathogen-associated molecular patterns (PAMPs) represents an early initiating event, triggering innate immune responses in keratinocytes and dendritic cells (DCs). Upon DAMP or PAMP recognition, keratinocytes produce a wide array of pro-inflammatory cytokines, including IFN-β, IL-1β, IL-36, TNF-α, IL-6, and CXCL10, promoting the differentiation and recruitment of inflammatory T-cell subsets [4]. Th17 lymphocyte stimulation of keratinocytes further amplifies inflammation via enhanced secretion of TNF-α, IL-1, IL-6, and CXCL8/IL-8, sustaining the chronic inflammatory response characteristic of psoriasis [5].

Current therapies for inflammatory skin disorders, including psoriasis, often alleviate symptoms but may cause side effects or have limited efficacy, highlighting the need for safe and effective complementary or alternative approaches [6].

Marine-derived natural products have emerged as promising sources of bioactive compounds with antioxidant and anti-inflammatory properties. *Posidonia oceanica* (L.) Delile, a seagrass endemic to the Mediterranean Sea, is rich in polyphenols and other bioactive molecules with potent antioxidant activity. Previous studies in our laboratory demonstrated that a hydroalcoholic extract from *P. oceanica* leaves (POE) exhibits significant anti-inflammatory and antioxidant effects in various experimental models [7]. In our prior in vivo study using a mouse model of imiquimod-induced psoriasis-like skin inflammation, POE attenuated both clinical and histological features of psoriasis and significantly reduced expression of psoriatic pro-inflammatory cytokines, including IL-17A, IL-17F, IL-23, TNF-α, IFN-γ, and IL-2, as well as the inflammation-associated adipokine lipocalin-2 (LCN-2) [8]. These findings support the therapeutic potential of POE and provide a rationale for assessing its direct effects on keratinocytes, the key initiators of psoriatic inflammation.

In vitro models are essential for dissecting molecular mechanisms underlying keratinocyte-mediated inflammatory responses. Human immortalized keratinocytes, such as HaCaT cells, retain many biological characteristics of normal human keratinocytes [9] and respond to pro-inflammatory stimuli by producing cytokines and reactive oxygen species (ROS) [10,11,12]. Although HaCaT cells do not fully recapitulate all aspects of epidermal differentiation in vivo, they are stable and reproducible, making them a reliable system to study keratinocyte-mediated inflammatory mechanisms (Table S1, Supplementary Materials).

Stimulation of HaCaT cells with lipopolysaccharide (LPS), a bacterial endotoxin, is widely used as an in vitro model of psoriasis (Table S1, Supplementary Materials). LPS induces secretion of pro-inflammatory cytokines, including IL-1β, IL-6, TNF-α, and CXCL8/IL-8 [13,14], and upregulates inducible nitric oxide synthase 2 (NOS2), resulting in increased nitric oxide (NO) production [15]. NOS2 and NO are critical in the pathogenesis of psoriasis and other inflammatory skin disorders [16]. LPS binds to TLR4, which can also be activated by DAMPs [4], leading to chronic inflammation, oxidative stress, and epidermal barrier disruption. TLR4 signals via both MyD88-dependent and TIR-domain-containing adapter-inducing interferon-β (TRIF)-dependent pathways: the MyD88 pathway triggers rapid NF-κB and MAPK activation and pro-inflammatory cytokine expression, while the TRIF pathway mediates delayed NF-κB activation, amplifying inflammation [17]. Consistently, our previous studies showed that POE counteracts LPS-induced NF-κB activation in murine RAW264.7 macrophages by modulating ERK1/2 and Akt signaling cascades [18], highlighting its potential to interfere with LPS-driven inflammation.

Based on these premises, the present study aimed to elucidate the cellular mechanisms underlying the protective and anti-inflammatory effects of POE in keratinocytes, the primary initiators and amplifiers of psoriatic inflammation. Using LPS-stimulated HaCaT cells, we evaluated the effects of POE on cell proliferation, apoptosis, ROS production, transcriptional and secretory profiles of key psoriatic cytokines (IL-1β, IL-6, TNF-α, IL-8), and NOS2/NO expression. This approach allowed us to assess the anti-inflammatory potential of POE in keratinocyte-mediated psoriasis-relevant responses and provided the opportunity to explore its potential effects on keratinocyte proliferation and apoptosis.

## 2. Results

### 2.1. Determination of Safe Working Concentrations of POE and LPS

**Figure 1:**
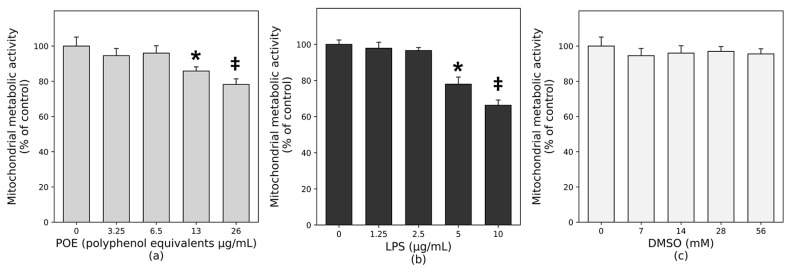
Effect of POE, LPS, DMSO, and their combination on HaCaT cell proliferation. HaCaT cells were treated for 12 h with increasing concentrations of POE, LPS, or DMSO. (**a**) HaCaT cells treated with increasing concentrations of POE (0–26 µg/mL, expressed as polyphenol equivalents); (**b**) HaCaT cells treated with increasing concentrations of LPS (0–10 µg/mL); (**c**) HaCaT cells treated with increasing concentrations of DMSO (0–56 mM; vehicle control). Mitochondrial metabolic activity was assessed using the MTT assay and expressed as a percentage of untreated cells. Data are presented as mean ± SD (n = 6). Statistical analysis was performed using one-way ANOVA followed by Tukey’s post hoc test. Different symbols (*, ‡) indicate statistically significant differences (*p* < 0.05). Bars that do not share a common symbol are significantly different from each other.

*Posidonia oceanica* extract (POE) was used in this study; its preparation and phytochemical characterization were performed in our previous studies, as described in Section 4. To identify the non-toxic threshold doses of POE and LPS for subsequent assays, HaCaT cell metabolic activity was assessed using the MTT assay, a colorimetric method that quantifies mitochondrial function. As shown in Figure 1, exposure to POE at concentrations up to 6.5 µg/mL (expressed as polyphenol equivalents; Figure 1a) or to LPS up to 2.5 µg/mL (Figure 1b) did not significantly affect metabolic activity compared with untreated controls. In contrast, higher concentrations of POE, such as 26 µg/mL polyphenol equivalents, induced a modest but significant reduction in metabolic activity (78.19 ± 3.10%, *p* < 0.05). Similarly, LPS at 5 and 10 µg/mL exerted marked cytotoxic effects, significantly reducing metabolic activity to 77.98 ± 3.94% and 66.29 ± 2.93%, respectively. Exposure to the vehicle DMSO did not significantly affect metabolic activity (Figure 1c).

Based on these findings, subsequent experiments were conducted using POE at 6.5 µg/mL polyphenol equivalents (dissolved in 14 mM DMSO as a vehicle), 2.5 µg/mL LPS, and 14 mM DMSO alone as a vehicle control.

### 2.2. POE Mitigates the Stimulatory Effect of LPS on HaCaT Cell Proliferation

**Figure 2:**
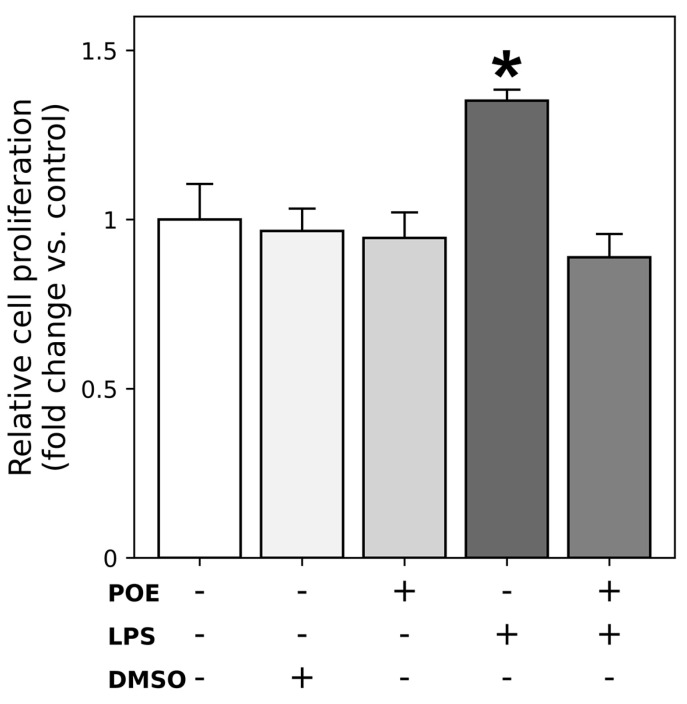
Effect of POE, LPS, DMSO, and their combination on HaCaT cell proliferation. Cell proliferation was assessed using the CyQUANT GR DNA-binding fluorescent dye. HaCaT cells were cultured under control conditions or treated for 12 h with LPS (2.5 µg/mL), POE (6.5 µg/mL polyphenol equivalents), DMSO (14 mM; vehicle for POE), or a combination of LPS + POE. The symbol + denotes the presence of the treatment, whereas − denotes its absence. Cell proliferation is expressed as fold change relative to control cells. Data are presented as mean ± SD (n = 6). Statistical analysis was performed using one-way ANOVA followed by Tukey’s post hoc test for multiple comparisons. The bar marked with * is significantly different from all other groups (*p* < 0.05). The remaining groups do not differ significantly from each other. The corresponding raw data expressed as arbitrary fluorescence units (AU) are reported in the Supplementary Materials (Figure S1).

Cell proliferation was assessed using the green fluorescent nucleic acid–binding CyQUANT dye, which quantifies cell number based on DNA content. As shown in Figure 2, treatment with LPS (2.5 µg/mL) significantly increased HaCaT cell proliferation, resulting in an approximately 1.4-fold increase compared with control cells (Figure 2; fold change vs. control: 1.35 ± 0.03, *p* < 0.05). In contrast, exposure to POE alone (6.5 µg/mL polyphenol equivalents) did not significantly affect cell proliferation (Figure 2; fold change vs. control: 0.95 ± 0.08, *p* > 0.05). Treatment with DMSO (14 mM), used as the vehicle for POE, had no effect on HaCaT cell proliferation. Instead, the POE co-treatment in LPS-stimulated cells significantly attenuated the LPS-induced proliferative response, restoring cell proliferation to levels comparable to those of untreated control cells (Figure 2; fold change vs. control: 0.89 ± 0.07, *p* > 0.05), indicating that POE attenuates the proliferative effect of LPS on HaCaT cells. The corresponding raw data are reported in the Supplementary Materials (Figure S1), where cell proliferation is expressed as arbitrary fluorescence units, reflecting fluorescence intensity measured using the CyQUANT assay.

### 2.3. Analyses of Apoptosis During LPS and POE Stimulation

**Figure 3:**
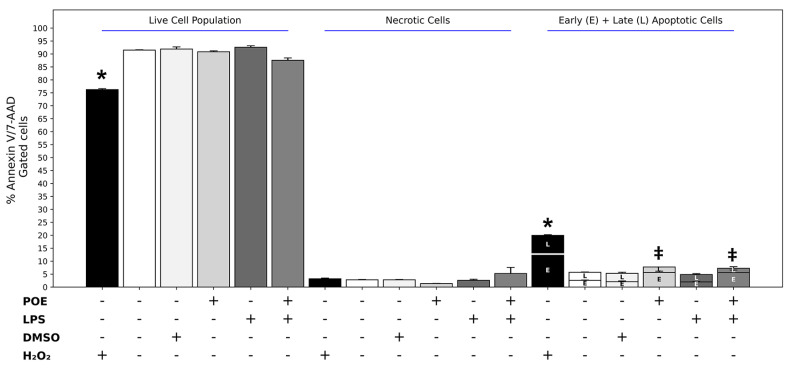
Analysis of apoptosis and necrosis in HaCaT cells following LPS and POE stimulation. Apoptosis and necrosis were assessed by flow cytometry using Annexin V/7-AAD staining. Quantitative analysis shows the percentage of live, necrotic, early apoptotic, and late apoptotic cells. The first six groups represent live cells, the next six represent necrotic cells, and the last six represent apoptotic cells, displayed as stacked bars separating early (E) and late (L) apoptosis. Treatments are H_2_O_2_, Control, DMSO, POE, LPS, and LPS + POE. The symbol + denotes the presence of the treatment, whereas − denotes its absence. Data are presented as mean ± SD (n = 4). Statistical analysis was performed using one-way ANOVA followed by Tukey’s post hoc test. Different symbols (*, ‡) indicate statistically significant differences (*p* < 0.05). Bars that do not share at least one common symbol are significantly different from each other. Representative Annexin V/7-AAD dot plots are provided in the Supplementary Materials (Figure S2).

Apoptosis was evaluated by flow cytometry using Annexin V/7-AAD staining. Representative Annexin V/7-AAD dot plots are shown in the Supplementary Materials (Figure S2a–f), while quantitative analyses of necrosis and apoptosis (early + late apoptosis) are summarized in Figure 3. Untreated control cells exhibited low basal levels of both necrosis and apoptosis (Figure 3; necrosis: 2.6 ± 0.42%, apoptosis: 4.8 ± 0.21%).

Similar values were observed in HaCaT cells exposed to LPS (2.5 µg/mL) (Figure 3; necrosis: 2.8 ± 0.14%, apoptosis: 5.7 ± 0.01%), indicating that LPS at this concentration did not induce apoptotic or necrotic effects.

In contrast, exposure of HaCaT cells to POE (6.5 µg/mL, expressed as polyphenol equivalents) resulted in a moderate but significant increase in apoptosis compared with control cells, without affecting necrosis (Figure 3; necrosis: 1.3 ± 0.07%, apoptosis: 7.75 ± 0.40%, *p* < 0.05). This pro-apoptotic effect was markedly lower than that observed in positive control cells treated with H_2_O_2_ (Figure 3; necrosis: 3.20 ± 0.28%, apoptosis: 19.95 ± 0.35%, *p* < 0.05).

Treatment with DMSO (14 mM), used as the vehicle control for POE, did not significantly affect apoptosis or necrosis, which remained comparable to control levels (Figure 3; necrosis: 2.8 ± 0.14%, apoptosis: 5.3 ± 0.70%, *p* > 0.05).

Co-treatment of HaCaT cells with LPS and POE did not significantly modify the pro-apoptotic effect induced by POE alone and did not result in a statistically significant increase in necrosis (Figure 3; necrosis: 5.2 ± 0.7%, apoptosis: 7.2 ± 1.6%, *p* > 0.05).

### 2.4. POE Reduces the ROS Increase Triggered by LPS Stimulation

**Figure 4:**
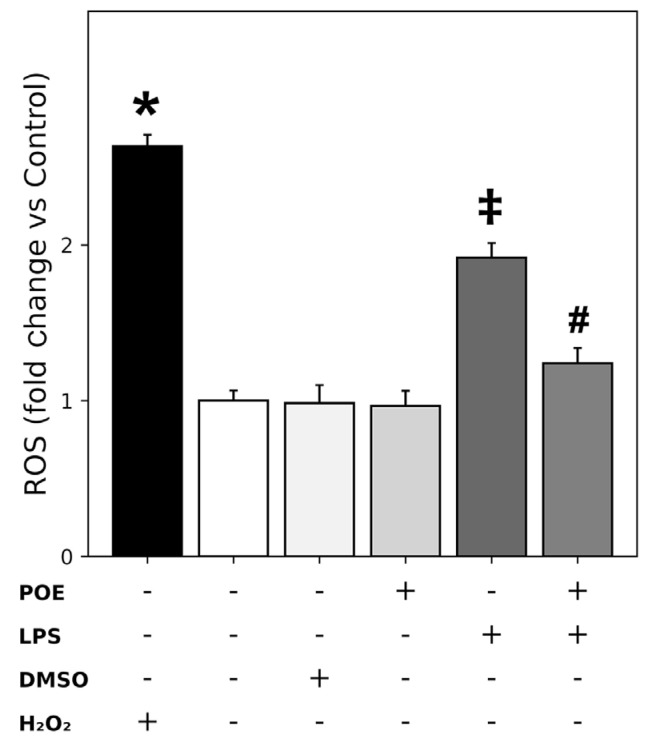
Detection of ROS levels in HaCaT cells by H_2_DCF-DA assay. HaCaT cells were treated for 12 h with H_2_O_2_ (200 µM, positive control), LPS (2.5 µg/mL), POE (6.5 µg/mL polyphenol equivalents), DMSO (14 mM, vehicle for POE), or a combination of LPS + POE. The symbol + denotes the presence of the treatment, whereas − denotes its absence. ROS production is shown as fold change relative to untreated control cells. Raw normalized fluorescence data (DCF-DA/MTT), are provided in the Supplementary Materials (Figure S3). Data are presented as mean ± SD (n = 6). Statistical significance was assessed by one-way ANOVA followed by Tukey’s post hoc test. Different symbols (*, ‡, #) indicate statistically significant differences (*p* < 0.05). Bars that do not share at least one common symbol are significantly different from each other.

Evaluation of ROS levels using the 2′,7′-dichlorodihydrofluorescein diacetate (H_2_DCF-DA) assay revealed that LPS stimulation (2.5 µg/mL) of HaCaT cells induced a significant but moderate increase in ROS compared with control cells (Figure 4; fold change vs. control: 1.91 ± 0.09, *p* < 0.05), which was lower than the strong increase triggered by H_2_O_2_ (200 µM), used as a positive control (Figure 4; fold change vs. control: 2.6 ± 0.07, *p* < 0.05). Treatment with POE alone (6.5 µg/mL polyphenol equivalents) or with the vehicle DMSO (14 mM) did not significantly affect ROS levels compared with control cells.

However, co-treatment with POE significantly attenuated the LPS-induced ROS increase, although a modest elevation remained (Figure 4; fold change vs. control: 1.24 ± 0.09, *p* < 0.05). Raw normalized fluorescence data for the ROS measurements are provided in the Supplementary Materials (Figure S3).

### 2.5. POE Reduces the LPS-Induced Nitric Oxide Secretion

**Figure 5:**
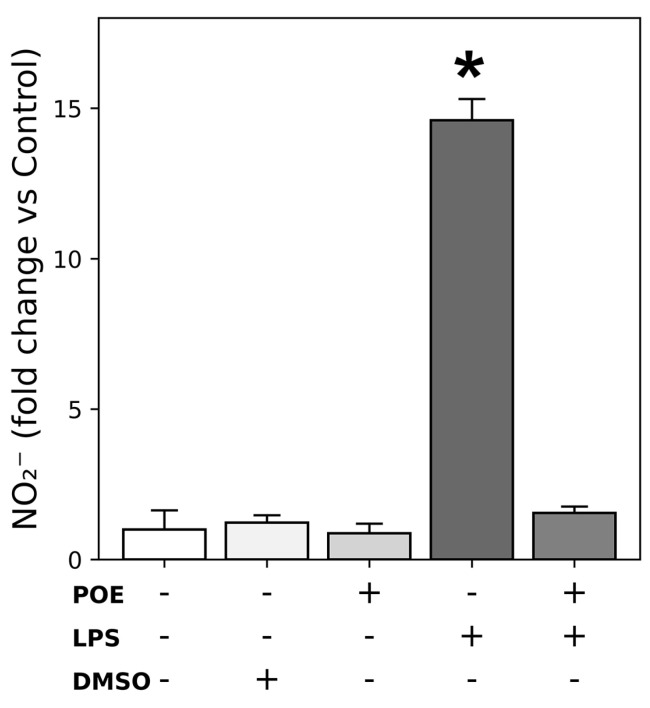
Effects of POE on nitric oxide (NO) secretion. HaCaT cells were stimulated for 12 h with LPS (2.5 µg/mL), POE (6.5 µg/mL polyphenol equivalents), DMSO (14 mM; vehicle for POE), or a combination of LPS + POE. The symbol + denotes the presence of the treatment, whereas − denotes its absence. Nitrite (NO_2_^−^), the stable metabolite of NO, was quantified in the culture medium using the Griess colorimetric assay. Fold changes in NO_2_^−^ levels relative to untreated control cells are shown. NO_2_^−^ levels expressed in nmol/10^6^ cells are reported in the Supplementary Materials (Figure S4). Data are presented as mean ± SD (n = 2). Statistical analysis was performed using one-way ANOVA followed by Tukey’s post hoc test. The bar marked with * is significantly different from all other groups (*p* < 0.05). The remaining groups do not differ significantly from each other.

Nitric oxide (NO) release following LPS stimulation (2.5 µg/mL) of HaCaT cells was quantified by measuring the accumulation of nitrite (NO_2_^−^), a stable and quantifiable metabolite of NO, using the Griess colorimetric assay. LPS stimulation markedly increased NO_2_^−^ levels compared with unstimulated control cells resulting in an approximately fourteen-fold increase (Figure 5; 14.60 ± 0.70-fold vs. control cells, *p* < 0.05).

Co-treatment of LPS-stimulated HaCaT cells with POE (6.5 µg/mL polyphenol equivalents) significantly reduced LPS-induced NO_2_^−^ secretion, restoring levels to values comparable with control cells (Figure 5; fold increase 1.5 ± 0.2, *p* < 0.05 vs. LPS-stimulated cells).

Treatment with POE alone or with the vehicle DMSO (14 mM, corresponding to the vehicle concentration for POE) did not significantly affect nitrite levels compared with untreated control cells.

NO_2_^−^ levels expressed as nmol/10^6^ cells are reported in the Supplementary Materials (Figure S4).

Overall, these findings demonstrate that POE inhibits LPS-induced NO production in HaCaT cells, further supporting its anti-inflammatory activity in this keratinocyte model.

### 2.6. POE Reduces LPS-Induced Cytokine Secretion in HaCaT Cells

**Figure 6:**
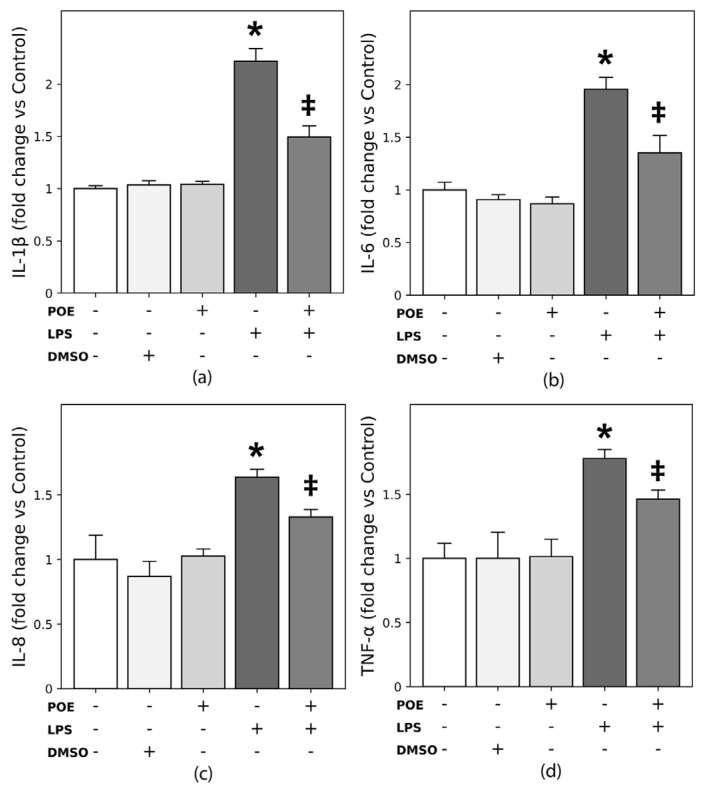
Effects of POE on cytokine secretion during LPS stimulation of HaCaT cells. HaCaT cells were stimulated for 12 h with LPS (2.5 µg/mL), POE (6.5 µg/mL polyphenol equivalents), DMSO (14 mM; vehicle control), or a combination of LPS + POE. The symbol + denotes the presence of the treatment, whereas − denotes its absence. Secretion of IL-1β, IL-6, IL-8, and TNF-α is shown as fold change relative to untreated control cells. Cytokine levels expressed in pg/10^6^ cells are reported in the Supplementary Materials (Figure S5). (**a**) IL-1β fold change relative to untreated control; (**b**) IL-6 fold change relative to untreated control; (**c**) IL-8 fold change relative to untreated control; (**d**) TNF-α fold change relative to untreated control. Data represent mean ± SD (n = 4). Statistical analysis was performed using one-way ANOVA followed by Tukey’s post hoc test. Differences are considered statistically significant at *p* < 0.05. Different symbols (*, ‡) indicate statistically significant differences (*p* < 0.05). Bars that do not share at least one common symbol are significantly different from each other.

The secretion of inflammatory cytokines following LPS stimulation (2.5 µg/mL) of HaCaT cells was quantified by ELISA for IL-1β, IL-6, IL-8, and TNF-α in the culture medium. LPS treatment markedly increased the secretion of IL-1β and IL-6, with fold increases of 2.21 ± 0.12 and 1.95 ± 0.11, respectively compared with unstimulated control cells (*p* < 0.05; Figure 6a,b). LPS also enhanced the secretion of IL-8 and TNF-α, with fold increases of 1.60 ± 0.06 and 1.77 ± 0.07, respectively (*p* < 0.05; Figure 6c,d), although these increases were more modest and did not reach the two-fold induction observed for IL-1β and IL-6.

Exposure of LPS-stimulated cells to POE (6.5 µg/mL polyphenol equivalents) significantly attenuated these elevations, resulting in fold increases of 1.49 ± 0.10 for IL-1β, 1.35 ± 0.16 for IL-6, 1.28 ± 0.05 for IL-8, and 1.46 ± 0.07 for TNF-α (*p* < 0.05 vs. LPS-stimulated cells), bringing cytokine levels close to those of untreated control cells.

Treatment with POE alone, or with the vehicle DMSO (14 mM, the concentration used for POE), did not significantly affect cytokine secretion compared with control cells.

Cytokine levels expressed as pg/10^6^ cells for all targets are reported in the Supplementary Materials (Figure S5).

Overall, these findings indicate that POE effectively mitigates the LPS-induced inflammatory cytokine response in HaCaT cells.

### 2.7. POE Suppresses Cytokine and Nitric Oxide Synthase 2 Expression Induced by LPS Stimulation

**Figure 7:**
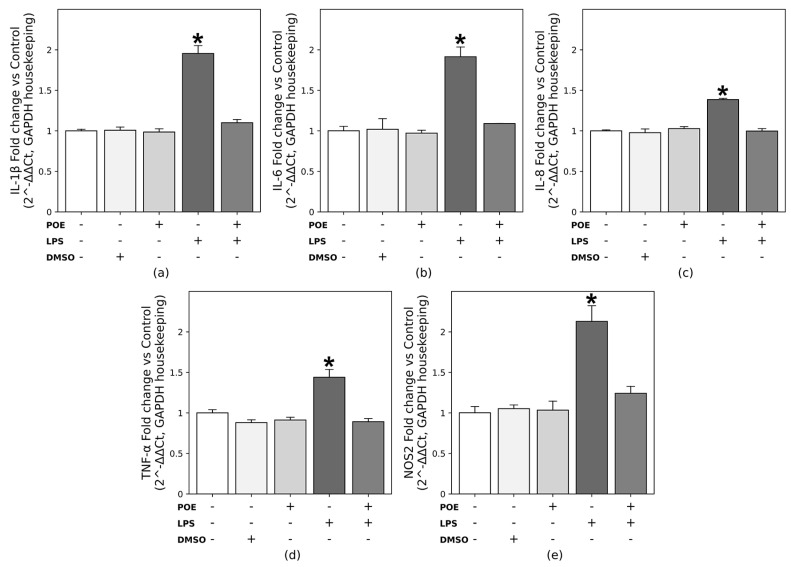
Effects of POE on mRNA expression of pro-inflammatory mediators in LPS-stimulated HaCaT cells. HaCaT cells were treated for 12 h with one of the following conditions: LPS (2.5 µg/mL), POE (6.5 µg/mL polyphenol equivalents), DMSO (14 mM; vehicle control), or a combination of LPS + POE. The symbol + denotes the presence of the treatment, whereas − denotes its absence. mRNA expression levels of *IL-1β* (**a**), *IL-6* (**b**), *IL-8* (**c**), *TNF-α* (**d**) and nitric oxide synthase 2 (*NOS2*) (**e**) were quantified by qRT-PCR and expressed as fold change relative to untreated control cells using the 2^−ΔΔCt^ method. Data are presented as mean ± SD (n = 3). Statistical analysis was performed using one-way ANOVA followed by Tukey’s post hoc test for multiple comparisons. The bar marked with * is significantly different from all other groups (*p* < 0.05). The remaining groups do not differ significantly from each other.

The gene expression levels of *IL-1β*, *IL-6*, *IL-8*, *TNF-α* and nitric oxide synthase 2 (*NOS2*) were quantified by qRT-PCR. As shown in Figure 7, stimulation of HaCaT cells with LPS (2.5 µg/mL) markedly upregulated the transcription of pro-inflammatory cytokines and *NOS2*. Specifically, *IL-1β* and *IL-6* mRNA levels nearly doubled compared with untreated control cells (Figure 7a, 1.95 ± 0.09-fold; Figure 7b, 1.91 ± 0.11-fold, respectively; *p* < 0.05). Similarly, *NOS2* mRNA expression was approximately two-fold higher than in unstimulated cells (Figure 7e, 2.12 ± 0.19-fold; *p* < 0.05).

More moderate but statistically significant increases were also observed for *IL-8* and *TNF-α* (Figure 7c, 1.38 ± 0.01-fold; Figure 7d, 1.44 ± 0.09-fold, respectively; *p* < 0.05), confirming that LPS activates a pro-inflammatory transcriptional program in HaCaT keratinocytes.

Exposure of LPS-stimulated HaCaT cells to POE (6.5 µg/mL polyphenol equivalents) restored the expression of all measured cytokines and *NOS2* to levels comparable to those of untreated controls, demonstrating that POE effectively counteracts LPS-induced cytokine overexpression and exerts a clear anti-inflammatory effect at the transcriptional level.

Treatment with POE alone or with the vehicle DMSO (14 mM, the concentration used for POE) did not significantly affect cytokine or *NOS2* expression compared with control cells. These findings are consistent with the results obtained from the analyses of cytokine and NO release.

## 3. Discussion

Several chronic inflammatory skin disorders, including atopic dermatitis, hidradenitis suppurativa, and psoriasis, are driven not only by dysregulated adaptive immune responses but also by aberrant activation of epidermal keratinocytes. In response to danger signals, keratinocytes initiate both protective and immunopathological reactions that critically contribute to disease onset and persistence [3,19]. Keratinocytes express a broad repertoire of TLRs, enabling them to recognize pathogen-associated molecular patterns (PAMPs) derived from microbial structures, such as LPS, as well as danger-associated molecular patterns (DAMPs), including host DNA and other endogenous molecules [20,21]. Engagement of TLRs triggers intracellular signaling cascades that converge on NF-κB and other transcription factors, promoting the expression of cytokines, chemokines, and co-stimulatory molecules involved in innate and adaptive immune responses.

Although the psoriatic skin microbiota is predominantly associated with Gram-positive bacteria, recent evidence indicates that lesional areas of inverse psoriasis display a higher abundance of Gram-negative bacteria compared with psoriasis vulgaris and healthy skin [22,23]. These findings support a role for Gram-negative bacteria in sustaining specific subtypes of psoriatic inflammation and provide a rationale for investigating Gram-negative-derived inflammatory stimuli in keratinocyte-based models.

In vitro, human HaCaT keratinocytes stimulated with LPS, a prototypical PAMP derived from Gram-negative bacteria, are widely used to study psoriasis-related inflammatory mechanisms. Canonical LPS signaling requires formation of a complex with LPS-binding protein (LBP) and CD14, which interacts with TLR4 and its co-receptor MD-2 [24,25]. HaCaT cells express CD14 and TLR4 but lack MD-2 [26]; nonetheless, LPS can still trigger inflammatory responses through alternative receptor complexes, including HSP70/HSP90, CXCR4, and GDF5 [27,28]. These pathways support the use of LPS-stimulated HaCaT cells as a simplified yet relevant in vitro model to investigate keratinocyte-intrinsic inflammatory responses, complementary to cytokine cocktails (e.g., M5) that reproduce adaptive immune-mediated signaling [29].

In our previous in vivo study, oral administration of the hydroalcoholic extract from *Posidonia oceanica* leaves (POE) significantly attenuated imiquimod-induced psoriasis-like inflammation in mice, reducing the expression of key psoriatic cytokines, including IL-17, IL-23, IFN-γ, IL-2, TNF-α, and lipocalin-2 [8]. Building on these findings, the present study focused on the cellular effects of POE on keratinocytes as primary responders in psoriasis, assessing its ability to modulate LPS-induced inflammatory, oxidative, and apoptotic responses in HaCaT cells. This approach complements our previous in vivo observations and explores the molecular mechanisms underlying POE’s anti-inflammatory activity.

In this study we show tthat non-cytotoxic concentrations of LPS and POE exert biologically relevant effects. LPS (2.5 µg/mL) promoted keratinocyte hyperproliferation without affecting mitochondrial metabolic activity or inducing apoptosis, whereas POE exerted anti-proliferative effects and induced a mild, non-cytotoxic increase in apoptosis (~8%). The absence of a pro-apoptotic effect of LPS aligns with previous reports indicating that low concentrations preferentially induce proliferative and inflammatory responses rather than programmed cell death [30,31]. LPS signaling activates NF-κB, MAPKs, and PI3K/Akt pathways, promoting cell survival and resistance to apoptosis, which parallels the hyperproliferative phenotype of psoriatic epidermis and supports the validity of LPS-stimulated HaCaT cells as a model of keratinocyte alterations in psoriasis [32,33,34,35].

In this context, the mild pro-apoptotic effect induced by POE, while not associated with overt cytotoxicity, may be of potential interest in psoriasis pathophysiology. Psoriatic keratinocytes are characterized not only by hyperproliferation but also by relative resistance to apoptosis, contributing to epidermal thickening and altered tissue homeostasis. Moreover, psoriasis requires lifelong management, and increasing attention has been directed toward the potential long-term risk of skin neoplasia associated with chronic inflammation and immune dysregulation intrinsic to the disease, as well as with prolonged systemic or biologic therapies [36,37]. Within this framework, POE’s ability to partially restore the proliferation/apoptosis balance through a controlled, mild induction of apoptosis could represent a biologically relevant feature. By counteracting apoptosis resistance without inducing excessive cell death, POE may help maintain epidermal homeostasis and potentially limit pro-tumorigenic signals linked to chronic inflammatory microenvironments. Although speculative, this possibility warrants further investigation, particularly in long-term models addressing keratinocyte transformation, genomic stability, and tumor-related signaling pathways.

In our study, LPS stimulation induced moderate increases in intracellular ROS levels, along with activation of transcription and secretion of pro-inflammatory cytokines IL-1β, IL-6, IL-8, and TNF-α. LPS also caused moderate upregulation of *NOS2* expression, resulting in a corresponding increase in nitric oxide (NO) production. This effect was associated with a limited enhancement of IL-8 and TNF-α secretion (less than two-fold vs. control), which are known to induce NO production [38,39]. Moderate, cytokine-dependent NO release may contribute to HaCaT cell proliferation, whereas higher NO concentrations trigger cell-cycle arrest [40]. Similarly, moderate ROS increases under non-cytotoxic LPS conditions may support proliferation without activating pro-apoptotic signaling, which is typically associated with excessive ROS. These findings are consistent with previous reports showing that low ROS levels promote proliferation, whereas high ROS levels induce apoptosis and cytotoxicity [41,42,43].

Under basal conditions, POE exposure did not induce measurable changes in intracellular oxidative status in keratinocytes, although a slight increase in apoptotic cells was observed. This dissociation suggests that the pro-apoptotic effect observed in the absence of inflammatory stimulation is unlikely to be primarily driven by oxidative imbalance and may involve alternative regulatory mechanisms of programmed cell death. Indeed, apoptosis can proceed independently of ROS through activation of extrinsic death receptor pathways, including Fas cell surface death receptor (Fas)- or TNF-mediated signaling, or via mitochondrial mechanisms leading to cytochrome c release and caspase-9 activation.

In contrast, under LPS stimulation, POE reduced intracellular oxidant species levels, supporting a protective effect against inflammation-associated redox disturbances. Considering the high content of (+)-catechin, a compound well recognized for its radical scavenging capacity, this dual behavior suggests that POE acts as a modulator of cellular redox balance, particularly under pro-inflammatory conditions, rather than as a constitutive antioxidant independent of context. Collectively, these findings indicate a context-dependent biological profile of POE, characterized by mild pro-apoptotic activity under basal conditions and preservation of redox homeostasis during inflammatory stress. Further studies are warranted to clarify the molecular pathways underlying this apparent ROS-independent apoptotic effect, including death receptor signaling, mitochondrial mediators, and caspase activation dynamics.

These observations also suggest that POE may influence endogenous systems responsible for maintaining oxidative balance. Beyond direct radical scavenging, it may modulate enzymatic antioxidant defenses, such as superoxide dismutase, catalase, and glutathione peroxidase, as well as upstream transcriptional regulators of cytoprotective gene expression. In psoriasis, the close interplay between oxidative imbalance and inflammatory signaling represents a central pathogenic feature.

The functional interaction between redox-sensitive transcription factors and pro-inflammatory pathways constitutes a regulatory hub coordinating cellular adaptation to stress and immune responses. NF-κB drives transcription of cytokines, chemokines, and adhesion molecules, whereas redox-responsive factors such as Nuclear factor erythroid 2–related factor 2 (Nrf2), Activator protein 1 (AP-1), and Hypoxia-inducible factor 1 alpha (HIF-1α) regulate genes involved in antioxidant defense, detoxification, and adaptation to oxidative or metabolic stress [44]. The balance between these pathways determines whether cells mount protective responses or progress toward inflammation-driven damage. Understanding how POE modulates this network may provide critical insight into its ability to integrate antioxidant and anti-inflammatory effects in keratinocytes under both basal and inflammatory conditions.

Consistent with this, POE treatment normalized LPS-induced upregulation of cytokine mRNA levels and partially reduced cytokine secretion. The persistence of low-level secretion likely reflects post-transcriptional regulation and differences in protein half-life [45]. By inhibiting IL-1β and IL-6, POE may attenuate early keratinocyte-derived signals that promote Th17 recruitment and inflammation [46,47]. Concurrent inhibition of IL-8 and TNF-α, together with normalization of NOS2 expression and NO production, likely contributed to the anti-proliferative effects of POE on keratinocytes. These in vitro findings complement our previous in vivo evidence, reinforcing the protective role of POE in psoriatic inflammation [8].

Although intracellular signaling was not directly assessed in this study, previous work from our laboratory demonstrated that POE inhibits NF-κB activation and modulates ERK1/2 and Akt pathways in macrophages [18], suggesting that similar mechanisms may operate in keratinocytes. Future studies will aim to dissect these signaling pathways in detail, focusing on the context-dependent roles of NF-κB and its crosstalk with redox-sensitive transcription factors such as Nrf2, AP-1, and HIF-1α, to clarify the molecular mechanisms through which POE regulates keratinocyte proliferation, apoptosis, and redox homeostasis.

In conclusion, POE exerts antioxidant, anti-inflammatory, and mild pro-apoptotic effects in LPS-stimulated HaCaT keratinocytes, counteracting keratinocyte hyperactivation, reducing oxidative stress and pro-inflammatory cytokine expression, and restoring proliferation–apoptosis balance. Beyond its anti-inflammatory properties, its context-dependent modulation of apoptosis and redox signaling may have additional implications for long-term epidermal homeostasis in chronic inflammatory settings. Together with our previous in vivo findings, these results support the therapeutic potential of POE as a marine-derived bioactive phytocomplex for complementary management of psoriasis and other inflammatory skin disorders, providing a rationale for further mechanistic and long-term safety investigations.

## 4. Materials and Methods

### 4.1. Chemicals and Reagents

Unless otherwise indicated, all chemicals and reagents were obtained from Sigma-Aldrich (Merck KGaA, Darmstadt, Germany).

### 4.2. Cell Line and Culture Conditions

In this study, the HaCaT cell line, consisting of immortalized human keratinocytes (Cytion GmbH, formerly Cell Line Service, Eppelheim, Germany, catalog no. 300493), was used. Cells were maintained at 37 °C in a humidified atmosphere with 5% CO_2_, using high-glucose Dulbecco’s modified Eagle’s medium (DMEM) as the standard culture medium (DMEM High Glucose Cat. No. 11965092, Gibco™, Thermo Fisher Scientific Inc., Monza, Italy), containing 25 mM D-glucose, 4 mM L-glutamine, 44.0 mM NaHCO_3_, and 1.8 mM calcium chloride, and supplemented with an additional 1 mM sodium pyruvate (Cat. No. 11360070, Gibco™, Thermo Fisher Scientific Inc., Monza, Italy) and 10% low-endotoxin, low-hemoglobin, heat-inactivated FBS (56 °C, 30 min) (Fetal Bovine Serum, Premium Plus, Cat. No. A5669701, Gibco™, Thermo Fisher Scientific Inc., Monza, Italy).

Once cultures reached approximately 80% confluence (subconfluent cultures), cells were detached using TrypLE™ Express (TrypLE™ Express Enzyme 1×, no phenol red, Cat. No. 12604039, Gibco™, Thermo Fisher Scientific Inc., Monza, Italy), collected, centrifuged for 10 min at 1000× *g* rpm, and resuspended in complete medium. Cells were subsequently subcultured by seeding into new culture plates at a density of 18 × 10^3^ cells/cm^2^. All experiments were performed using cells between passages 10 and 25. On the day prior to each experimental procedure, the culture medium was replaced with DMEM containing 1% FBS (serum-deprived medium).

### 4.3. Hydroalcoholic Extract of P. oceanica

The hydroalcoholic extract from *P. oceanica* leaves (POE) was prepared as previously described [8]. Briefly, POE obtained from 4 g of dried *P. oceanica* leaves yielded 52 mg/mL of dry extract after resuspension in 1000 µL of DMSO. Since POE is mainly composed of polyphenols (approximately 88%), as previously reported [48], the concentrations of POE used in all subsequent experimental procedures were expressed as polyphenol equivalents. This approach allows the biological activity to be related specifically to the phenolic fraction and ensures consistency with previously published studies on *P. oceanica* extracts.

The total polyphenol content of POE, quantified using the Folin–Ciocalteu method, was 6.5 ± 1.29 mg/mL, expressed as gallic acid equivalents. Antioxidant activity, measured by the FRAP assay, and free radical scavenging capacity, assessed by the DPPH assay, were 0.98 ± 0.2 mg/mL and 1.3 ± 0.1 mg/mL of ascorbic acid equivalents, respectively.

**Figure 8:**
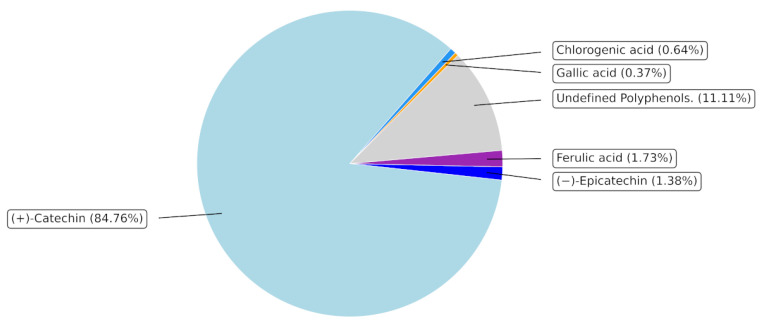
Polyphenolic composition of *Posidonia oceanica* leaves extract. POE, analyzed by UPLC, contains a well-defined phenolic fraction representing approximately 88% of the extract. (+)-Catechin is the predominant component (84.76%), while gallic acid, chlorogenic acid, (−)-epicatechin, and ferulic acid are present in smaller amounts. Undefined polyphenols account for 11.11%. Percentages indicate the proportion of each compound within the extract.

Additional details regarding the extraction procedure are provided in reference [49], and a comprehensive characterization of the major phytochemical components of POE is presented in Figure 8.

### 4.4. Identification of Non-Toxic Doses of LPS and POE

To identify the non-toxic threshold doses of LPS and POE, defined as the highest concentrations that did not significantly reduce mitochondrial metabolic activity, the effects of these treatments were evaluated using the colorimetric 3-(4,5-dimethylthiazol-2-yl)-2,5-diphenyltetrazolium bromide (MTT) assay. The MTT assay specifically measures mitochondrial activity, based on the ability of mitochondrial dehydrogenases in metabolically active cells to reduce the water-soluble yellow tetrazolium salt into insoluble purple formazan crystals. This reduction provides a quantitative readout of mitochondrial metabolic function and, indirectly, cell viability. Non-toxic concentrations were defined as those that did not significantly alter metabolic activity, indicating preservation of viable cells and absence of cytotoxicity.

Briefly, subconfluent HaCaT cells were seeded into 96-well plates at a density of 1 × 10^4^ cells/well and cultured overnight in serum-deprived medium prior to treatment. The medium was then replaced with serum-deprived medium containing either two-fold serial dilutions of LPS (ranging from 10 to 0.625 µg/mL; Lipopolysaccharide 500× solution, *Escherichia coli* serotype O26:B6, Cat. No. 00-4976-93, eBioscience™, Thermo Fisher Scientific Inc., Monza, Italy), two-fold serial dilutions of POE expressed as polyphenol equivalents (ranging from 26 ± 4.8 to 3.25 ± 0.6 µg/mL), or the corresponding DMSO concentrations used for POE to control for solvent-related effects (ranging from 0 to 56 mM). Untreated cells were used as controls.

After 12 h of incubation, the medium was removed, and cell monolayers were washed with phosphate-buffered saline (PBS). Subsequently, 100 µL/well of MTT solution (0.5 mg/mL) was added, and plates were incubated for 1 h at 37 °C in the dark. Cells were then washed with PBS and lysed with 100 µL/well of dimethyl sulfoxide (DMSO). The absorbance of the solubilized formazan was measured at 595 nm using a microplate reader (Model 550 Microplate Reader, Bio-Rad Laboratories, Inc., Hercules, CA, USA).

The 12-h treatment duration was selected to capture the acute cellular response to LPS and POE, taking into account the preceding 12-h pre-incubation in serum-deprived medium, while minimizing potential confounding effects of prolonged starvation, which could induce cellular stress, apoptosis, or metabolic alterations.

Mitochondrial metabolic activity was expressed as a percentage of untreated control cells. All experiments were performed in six replicate wells.

### 4.5. Cell Proliferation Assay

Once the minimal non-toxic concentrations of LPS and POE were identified, these doses were tested for their effects on cell proliferation using the CyQUANT^®^ Cell Proliferation Assay (Invitrogen, Cat. No. C35011, Thermo Fisher Scientific Inc., Monza, Italy), according to the manufacturer’s instructions. This assay is based on the binding of the fluorometric CyQUANT GR dye to cellular DNA, providing a quantitative measure of DNA content and, therefore, allowing evaluation of cell proliferation and DNA replication.

Briefly, HaCaT cells were detached from subconfluent cultures and seeded into 96-well plates at a density of 1 × 10^4^ cells/well in serum-deprived medium. After overnight incubation, the medium was replaced with serum-deprived medium alone (control, non-supplemented) or supplemented with LPS (at its minimal non-toxic concentration), POE (at its minimal non-toxic concentration), or LPS + POE (both agents at their minimal non-toxic concentrations). Additional wells containing DMSO at concentrations corresponding to the POE treatment were included to control for solvent-related effects.

After 12 h of incubation, 100 µL of CyQUANT^®^ 2X detection reagent were added to each well containing 100 µL of culture medium, and plates were incubated for 60 min at 37 °C. Fluorescence was then measured at an excitation wavelength of 485 nm and an emission wavelength of 538 nm using a Synergy™ 1H Multi-Mode Microplate Reader (fluorimeter) (BioTek Instruments, Inc., Winooski, VT, USA). Data were expressed either as fluorescence intensity or as fold change relative to control, and all experiments were performed in six replicate wells.

### 4.6. Flow Cytometric Analysis of Apoptosis

Apoptotic and necrotic cell populations were evaluated by flow cytometry to assess the impact of treatments on HaCaT keratinocyte viability. HaCaT cells (5 × 10^5^) were cultured overnight in serum-deprived medium and subsequently incubated for 12 h with the indicated concentrations of LPS, POE, or their combination (LPS + POE). As a positive control, cells were treated with 200 µM H_2_O_2_ for 2 h. Following treatment, cells were detached using TrypLE™ Express 1× (no phenol red; Cat. No. 12604039, Gibco™, Thermo Fisher Scientific Inc., Monza, Italy) and collected for analysis.

Cells were harvested along with the supernatants, centrifuged at 1000× *g*, and resuspended in 500 µL of 1× Annexin Binding Buffer (BD Pharmingen™ Annexin V Binding Buffer 10× concentrate Cat. No. 556454, BD Biosciences, San Jose, CA, USA). The cell suspension was then incubated with 5 µL of Annexin V-BV421 (BD Horizon™ BV421 Annexin V, Cat. No. 563973, BD Biosciences, San Jose, CA, USA) and 5 µL of 7-Amino-Actinomycin D (7-AAD) (BD Pharmingen™ 7-AAD, Cat. No. 559925, BD Biosciences, San Jose, CA, USA) at room temperature for 30 min in the dark.

Cell suspensions from quadruplicate samples for each experimental condition were analyzed by flow cytometry using a BD FACSCanto™ II Flow Cytometer (BD Biosciences, San Jose, CA, USA). Data were processed with BD FACSDiva™ v9.0 Software (BD Biosciences, San Jose, CA, USA), and a minimum of 10,000 events was collected per sample.

### 4.7. ROS Detection

To determine total ROS production in HaCaT cells following exposure to LPS, POE, or both, intracellular ROS levels were measured using the fluorescent probe 2′,7′-dichlorodihydrofluorescein diacetate (H_2_DCF-DA). Briefly, HaCaT cells were seeded into 96-well plates at a density of 1 × 10^4^ cells/well and incubated overnight in serum-deprived medium. Cell monolayers were then incubated for 12 h in serum-deprived medium either alone (control) or supplemented with the indicated minimal non-toxic concentrations of LPS, POE, or both. In addition, cells were stimulated with H_2_O_2_ (200 µM) for 2 h as a positive control.

At the end of the incubation, the medium was removed, monolayers were gently washed with PBS, and 100 µL/well of 10 µM H_2_DCF-DA in PBS was added. Cells were incubated for 1 h at 37 °C in the dark. Monolayers were then washed with PBS, and fluorescence was immediately measured at an excitation wavelength of 485 nm and an emission wavelength of 535 nm using a Synergy™ 1H Multi-Mode Microplate Reader (BioTek Instruments, Inc., Winooski, VT, USA).

ROS levels were normalized to cell metabolic integrity, as evaluated in replicate wells by the MTT assay, and data were expressed either as normalized fluorescence (arbitrary units) relative to MTT absorbance at 595 nm, or as fold change versus control. All experiments were performed in six replicate wells.

### 4.8. Assessment of Nitric Oxide Production

To evaluate the effect of POE on NO release during LPS stimulation, the accumulation of NO_2_^−^ in the culture medium was measured. Nitrite is a stable end product of NO, which is highly reactive and rapidly converted in the presence of oxygen and water. Therefore, nitrite provides an indirect estimation of NO production and can be detected and quantified photometrically using the Griess colorimetric reaction.

Briefly, subconfluent HaCaT cells were detached as described above, seeded into 24-well plates at a density of 2 × 10^5^ cells/well, and incubated overnight in serum-deprived medium. The medium was then replaced with 200 µL per well of serum-deprived DMEM without phenol red (DMEM High Glucose, no phenol red, Cat. No. 31053028, Gibco™, Thermo Fisher Scientific Inc., Monza, Italy), either alone (control) or supplemented with the indicated minimal non-toxic concentrations of LPS, POE, or their combination (LPS + POE). After 12 h of incubation, culture medium was collected and centrifuged at 500× *g* for 5 min at 4 °C to remove cellular debris, and cells from each well were detached and counted using a hemocytometer.

Aliquots of 50 µL of the clarified supernatants were mixed with an equal volume of Griess reagent and incubated at room temperature for 15 min. Nitrite concentrations were determined in nmol/mL using a standard curve prepared with sodium nitrite in the range of 0–65 nmol/mL, by measuring absorbance at 540 nm with a microplate reader (Model 550 Microplate Reader, Bio-Rad Laboratories, Inc., Hercules, CA, USA).

To normalize nitrite secretion to cell number, nitrite concentrations (nmol/mL) were converted to the total amount of nitrite per well and subsequently normalized to one million cells using the following formula:

Nitrite per 1,000,000 cells (nmol/10^6^ cells) = (Nitrite concentration in the supernatant (nmol/mL) × Supernatant volume (mL) ÷ Number of cells) × 1,000,000

Normalized values were used to compare nitrite secretion across experimental conditions.

### 4.9. Assessment of Cytokine Secretion by ELISA

To assess the effect of POE on IL-1β, IL-6, IL-8, and TNF-α secretion by HaCaT cells during LPS stimulation, cytokine levels were quantified by ELISA. Subconfluent HaCaT cells were detached as described above, seeded into 6-well plates at a density of 5 × 10^5^ cells/well, and incubated overnight in serum-deprived medium. The medium was then replaced with 2 mL per well of serum-deprived medium either alone (control) or supplemented with the indicated minimal non-toxic concentrations of LPS, POE, or their combination (LPS + POE). After 12 h of incubation, culture media were collected and centrifuged at 500 × *g* for 5 min at 4 °C to remove cellular debris, and cells from each well were detached and counted using a hemocytometer.

Aliquots of 50 µL of the clarified supernatants were subsequently analyzed in quadruplicate using the following ELISA kits, according to the manufacturers’ instructions: IL-1β, Human IL-1β High Sensitivity ELISA Kit (Cat. No. BMS224-2HS, Invitrogen, Thermo Fisher Scientific, Monza, Italy; analytical sensitivity 0.05 pg/mL; assay range 0.16–10.0 pg/mL); IL-6, Human IL-6 High Sensitivity ELISA Kit (Cat. No. BMS213-2HS, Invitrogen, Thermo Fisher Scientific, Monza, Italy; analytical sensitivity 0.03 pg/mL; assay range 0.08–5.0 pg/mL); IL-8, Human IL-8 Ultrasensitive ELISA Kit (Cat. No. KHC0084-2, Invitrogen, Thermo Fisher Scientific, Monza, Italy; analytical sensitivity 0.19 ng/mL; assay range 0.31–20 ng/mL); TNF-α, Human TNF-α Ultrasensitive ELISA Kit (Cat. No. KHC3014, Invitrogen, Thermo Fisher Scientific, Monza, Italy; analytical sensitivity <0.09 pg/mL; assay range 0.5–32 pg/mL).

Cytokine concentrations were calculated in pg/mL from standard curves by measuring absorbance at 450 nm using a microplate reader (Model 550 Microplate Reader, Bio-Rad Laboratories, Inc., Hercules, CA, USA). To normalize cytokine secretion to cell number, cytokine concentrations (pg/mL) were converted to the total amount of cytokine per well and subsequently normalized to one million cells using the following formula:

Cytokine per 1,000,000 cells (pg/10^6^ cells) = (Cytokine concentration in the supernatant (pg/mL) × Supernatant volume (mL) ÷ Number of cells) × 1,000,000

Normalized values were used to compare cytokine secretion across experimental conditions.

### 4.10. Cytokine and Nitric Oxide Synthase 2 Expression by qRT-PCR

To assess the effect of POE on *IL-1β*, *IL-6*, *IL-8*, *TNF-α*, and *NOS2* expression during LPS stimulation of HaCaT cells, qRT-PCR was performed. HaCaT cells were grown in serum-deprived medium, and after overnight incubation, the medium was replaced with serum-deprived medium either alone (control) or supplemented with the indicated minimal non-toxic concentrations of LPS, POE, or their combination (LPS + POE), as described above. After an additional 12 h of incubation, total RNA was extracted from 2 × 10^6^ cells using the RNeasy^®^ Mini Kit (Cat. No. 74106, Qiagen, Milan, Italy) according to the manufacturer’s instructions. RNA concentration and purity were assessed by measuring absorbance at 260 nm and the 260/280 nm ratio, respectively.

A total of 1 µg of RNA was reverse-transcribed into cDNA using the QuantiTect Reverse Transcription Kit (Cat. No. 205311, Qiagen, Hilden, Germany). qRT-PCR reactions were performed in triplicate for each target gene and for the housekeeping gene using a BIO-RAD C1000 Touch Thermal Cycler with CFX96 Real-Time System (Bio-Rad Laboratories, Inc., Hercules, CA, USA). Each reaction contained 50 ng of cDNA and PowerSYBR^®^ Green PCR Master Mix (Cat. No. A46109, Thermo Fisher Scientific, Monza, Italy). Primers commercially sourced from Origene Technologies Inc. (OriGene Technologies GmbH, Herford, Germany) are listed in Table S2 of the Supplementary Materials.

### 4.11. Statistical Analysis

All data are expressed as mean ± standard deviation (SD), and the number of samples analyzed for each group is indicated in the figure legends. Statistical differences among treatment groups were assessed using one-way ANOVA, followed by Tukey’s post hoc test for multiple comparisons. Analyses were performed using PSPP (version 2.0.0 64-bit, GNU Project, Free Software Foundation, Boston, MA, USA). Differences were considered statistically significant at *p* < 0.05.

## Data Availability

The data presented in this study are available on request from the corresponding author.

## Supplementary Materials

The following supporting information can be downloaded at: https://www.mdpi.com/article/10.3390/md24020085/s1, Figure S1: Raw fluorescence data for HaCaT cell proliferation following POE, LPS, DMSO, or combined treatment; Figure S2: Dot blot analysis of apoptosis and necrosis in HaCaT cells following LPS and POE stimulation; Figure S3: Raw normalized fluorescence data of ROS levels in HaCaT cells measured by H_2_DCF-DA assay; Figure S4: Raw data of Nitrite (NO_2_^−^) secretion (nmol/10^6^ cells) in HaCaT cells following POE treatment; Figure S5: Raw data of cytokine secretion (pg/10^6^ cells) in HaCaT cells during LPS stimulation following POE treatment; Table S1: Main references from the last 5 years using LPS-stimulated HaCaT cells as inflammatory and psoriatic models [50,51,52,53,54,55,56,57,58,59]; Table S2: Primer Sequences and Target Genes for qRT-PCR.

## Abbreviations

The following abbreviations are used in this manuscript:

AD	Atopic dermatitis
Akt	Protein kinase B, Ak is the mouse strain and T is transforming ability
ANOVA	Analysis of variance
AP-1	Activator protein 1
CD14	Cluster of differentiation 14
cDNA	Complementary DNA
CXCL10	C-X-C motif chemokine ligand 10
CXCL8/IL-8	C-X-C motif chemokine ligand 8/Interleukin-8
CXCR4	C-X-C chemokine receptor type 4
DAMPs	Damage-associated molecular patterns
DMSO	Dimethyl sulfoxide
DMEM	Dulbecco’s modified Eagle’s medium
DPPH	2,2-diphenyl-1-picrylhydrazyl
ELISA	Enzyme-linked immunosorbent assay
ERK1/2	Extracellular signal-regulated kinases 1 and 2
Fas	Fas cell surface death receptor also known as Cluster of Differentiation 95 (CD95) or Apoptosis antigen-1 (APO-1).
FBS	Fetal bovine serum
FRAP	Ferric reducing antioxidant power
GDF5	Growth/Differentiation Factor 5
H_2_O_2_	Hydrogen peroxide
H_2_DCF-DA	2′,7′-Dichlorodihydrofluorescein diacetate
HaCaT	Human adult (keratinocytes) propagated under low Ca^2+^ and elevated Temperature
HIF-1α	Hypoxia-inducible factor 1 alpha
HSP70	Heat shock protein 70
HSP90	Heat shock protein 90
IFN-β	Interferon beta
IFN-γ	Interferon gamma
IL-1α	Interleukin-1 alpha
IL-1β	Interleukin-1 beta
IL-2	Interleukin-2
IL-6	Interleukin-6
IL-8/CXCL8	Interleukin-8/C-X-C motif chemokine ligand 8
IL-17A	Interleukin-17A
IL-17F	Interleukin-17F
IL-22	Interleukin-22
IL-23	Interleukin-23
IMQ	Imiquimod
LBP	LPS-binding protein
LCN-2	Lipocalin-2
LPS	Lipopolysaccharide
MAPKs	Mitogen-activated protein kinases
MD-2	Myeloid differentiation factor 2
MyD88	Myeloid differentiation primary response 88
MTT	3-(4,5-dimethylthiazol-2-yl)-2,5-diphenyltetrazolium bromide
Nrf2	Nuclear factor erythroid 2–related factor 2
NF-κB	Nuclear factor kappa-light-chain-enhancer of activated B cells
NO	Nitric oxide
NO_2_^−^	Nitrite
NOS2 (iNOS)	Inducible nitric oxide synthase 2
PBS	Phosphate-buffered saline
PCR	Polymerase chain reaction
PI3K	Phosphoinositide 3-kinase
POE	Posidonia oceanica extract
*P. oceanica*	Posidonia oceanica
PAMPs	Pathogen-associated molecular patterns
RAW264.7	Raschke William 264.7 Murine macrophage cell line
RNA	Ribonucleic acid
ROS	Reactive oxygen species
Th1	Type 1 T helper lymphocytes
Th17	Type 17 T helper lymphocytes
TNF-α	Tumor necrosis factor alpha
TLRs	Toll-like receptors
TLR4	Toll-like receptor 4
UPLC	Ultra-performance liquid chromatography
Rpm	Revolutions per minute
