# Supplementary material for: Posidonia oceanica (L.) Delile as a Marine Anti-Inflammatory Modulator of Keratinocyte Inflammatory Responses Relevant to Psoriasis
Source: Mar Drugs. 2026 Feb 19;24(2):85. doi: 10.3390/md24020085 (PMC12941391; doi:10.3390/md24020085)
Supplement: Supplementary file 1 [file marinedrugs-24-00085-s001.zip › marinedrugs-4149151-supplementary.pdf]

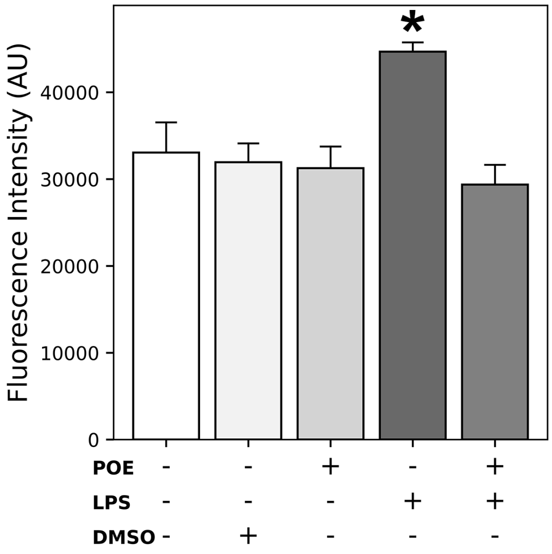

Figure S1. Raw fluorescence data for HaCaT cell proliferation following POE, LPS, DMSO, or combined treatment. Cell proliferation was assessed using the CyQUANT GR DNA-binding fluorescent dye. HaCaT cells were cultured under control conditions or treated for 12 h with LPS (2.5 µg/mL), POE (6.5 µg/mL polyphenol equivalents), DMSO (14 mM; vehicle for POE), or a combination of LPS + POE. The symbol + denotes the presence of the treatment, whereas − denotes its absence. Cell proliferation is shown as arbitrary fluorescence units (AU). Data are presented as mean ± SD (n = 6). Statistical analysis was performed using one-way ANOVA followed by Tukey's post hoc test for multiple comparisons. The bar marked with * is significantly different from all other groups (p < 0.05). The remaining groups do not differ significantly from each other.

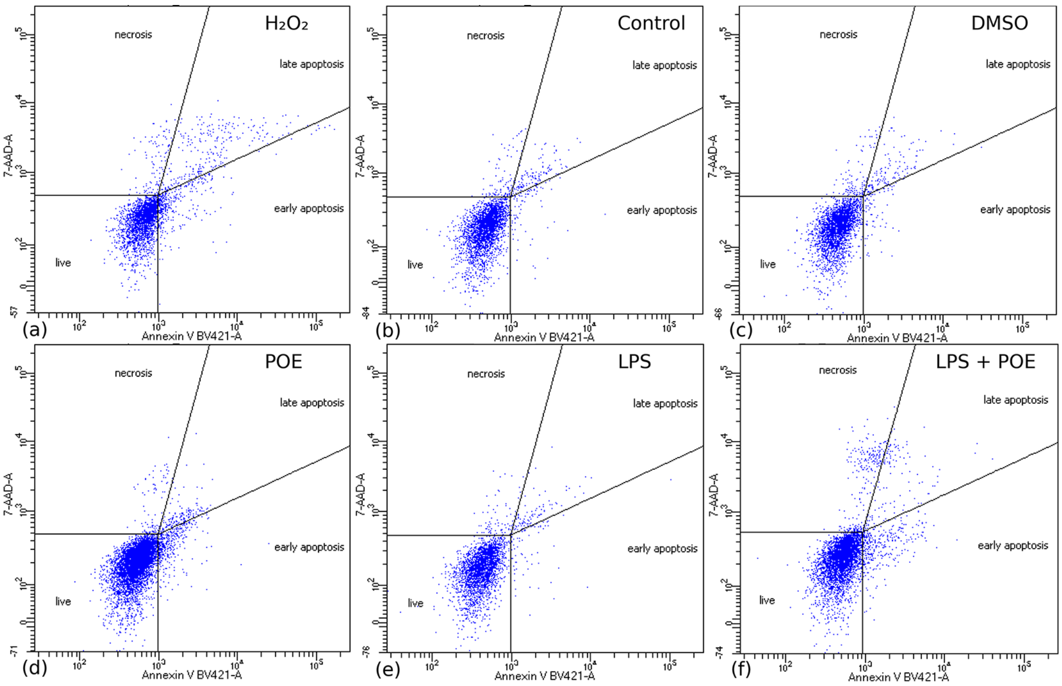

Figure S2. Dot blot analysis of apoptosis and necrosis in HaCaT cells following LPS and POE stimulation. Apoptosis and necrosis were assessed by flow cytometry using Annexin V/7-AAD staining. Representative dot plots are shown for (a) H_2O_2-treated cells (positive control), (b) untreated control cells, (c) DMSO-treated cells (14 mM, vehicle for POE), (d) POE-treated cells (6.5 µg/mL polyphenol equivalents), (e) LPS-treated cells (2.5 µg/mL), and (f) cells co-stimulated with LPS and POE.

Quadrant analysis distinguishes live, early apoptotic, late apoptotic, and necrotic populations. Data are representative of four independent experiments.

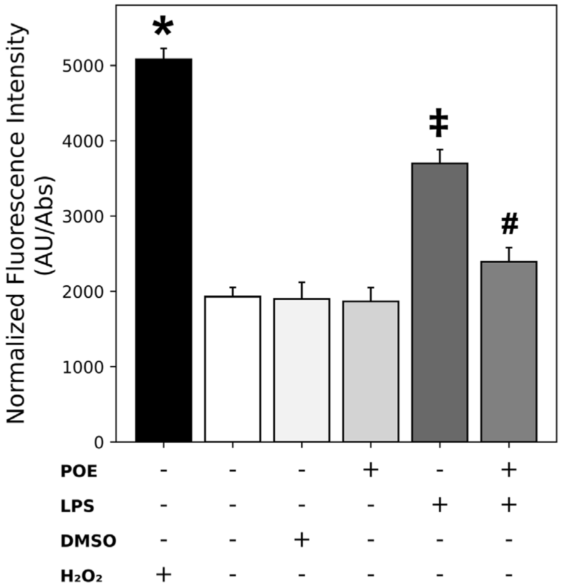

Figure S3. Raw normalized fluorescence data of ROS levels in HaCaT cells measured by H_2DCF-DA assay. HaCaT cells were treated for 12 h with H_2O_2 (200 μM, positive control), LPS (2.5 μg/mL), POE (6.5 μg/mL polyphenol equivalents), DMSO (14 mM, vehicle for POE), or a combination of LPS + POE. The symbol + denotes the presence of the treatment, whereas − denotes its absence. ROS production is shown as normalized fluorescence (DCF-DA/MTT, arbitrary units/absorbance. Statistical significance was assessed by one-way ANOVA followed by Tukey's post hoc test. Different symbols (*, ‡, #) indicate statistically significant differences ($p < 0.05$). Bars that do not share at least one common symbol are significantly different from each other.

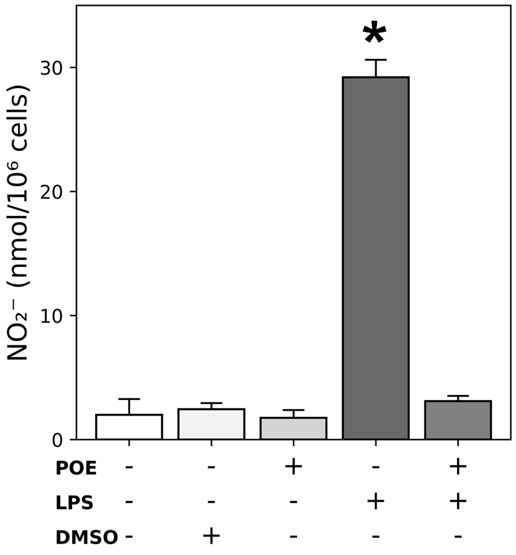

Figure S4. Raw data of Nitrite (NO_2^-) secretion (nmol/10^6 cells) in HaCaT cells following POE treatment. HaCaT cells were stimulated for 12 h with LPS (2.5 μg/mL), POE (6.5 μg/mL polyphenol equivalents), DMSO (14 mM; vehicle for POE), or a combination of LPS + POE. The symbol + denotes the presence of the treatment, whereas − denotes its absence. Nitrite (NO_2^-), the stable metabolite of NO, was quantified in the culture medium using the Griess colorimetric assay. Nitrite levels are expressed as nmol/10^6 cells. Data are presented as mean ± SD (n = 2). Statistical analysis was

performed using one-way ANOVA followed by Tukey's post hoc test. The bar marked with * is significantly different from all other groups ($p < 0.05$). The remaining groups do not differ significantly from each other.

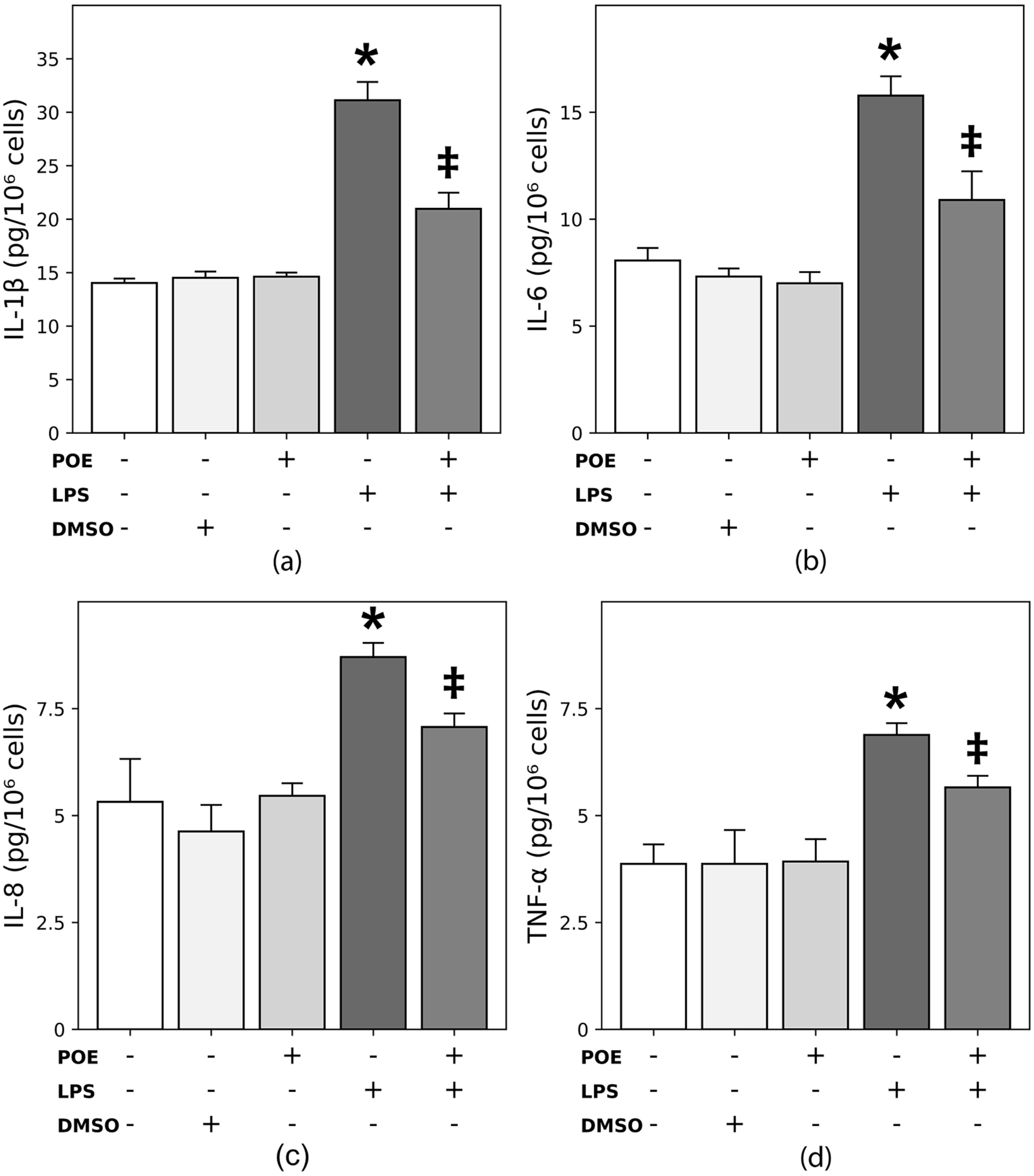

Figure S5. Raw data of cytokine secretion (pg/10⁶ cells) in HaCaT cells during LPS stimulation following POE treatment. HaCaT cells were stimulated for 12 h with LPS (2.5 µg/mL), POE (6.5 µg/mL polyphenol equivalents), DMSO (14 mM; vehicle control), or a combination of LPS + POE. The symbol + denotes the presence of the treatment, whereas − denotes its absence. Secretion of IL-1β, IL-6, IL-8, and TNF-α is shown as absolute concentrations (pg/10^6 cells) for (a) IL-1β, (b) IL-6, (c) IL-8, and (d) TNF-α. Data represent mean ± SD (n = 4). Statistical analysis was performed using one-way ANOVA followed by Tukey's post hoc test. Different symbols (*, ‡) indicate statistically significant differences ($p < 0.05$). Bars that do not share at least one common symbol are significantly different from each other.

Table S1. Main references from the last 5 years using LPS-stimulated HaCaT cells as inflammatory and psoriatic models.

Year	Reference	Main effect of LPS stimulation
2025	[50]	The effect of Adalimumab, a monoclonal antibody that inhibits tumor necrosis factor-alpha (TNF-α) used in psoriasis therapy, was evaluated on LPS-induced NF-κB–driven inflammation in HaCaT cells.
2024	[51]	LPS induces inflammatory phenotype (↑IL-1β, ↑IL-6, ↑NF-κB).
2023	[52]	LPS induces keratinocyte inflammation for anti-inflammatory evaluation.
2023	[53]	LPS triggers pro-inflammatory activation to test Etanercept effects.
2023	[54]	LPS induces inflammation, apoptosis, and proliferation changes in HaCaT.
2023	[55]	LPS (1 μg/mL) stimulates NO, IL-6, IL-1β, NF-κB/MAPK activation.
2022	[56]	LPS triggers inflammatory phenotype to test polysaccharide effects.
2021	[57]	LPS induces inflammatory stress in HaCaT cells.
2021	[58]	LPS arouses inflammatory damage (↓viability, ↑IL-6/TNF-α/COX-2).
2020	[59]	LPS induces inflammation e hyperproliferation in HaCaT cells.

All references listed in Table S1 are included in the main reference list of the publication

Table S2. Primer Sequences and Target Genes for qRT-PCR.

Gene product	Direction	Sequence (5′→3′)	Product size	Cat. No.
IL-1β	F	CCACAGACCTTCCAGGAGAATG	131 bp	HP200544
	R	GTGCAGTTCAGTGATCTACAGG		
IL-6	F	AGACAGCCACTCACCTCTTCAG	132 bp	HP200567
	R	TTCTGCCAGTGCCTCTTTGCTG		
IL-8	F	GAGAGTGATTGAGAGTGGACAC	112 bp	HP200551
	R	CACAACCCTCTGCACCCAGTTT		
TNF-α	F	CTCTTCTGCCTGCTGCACTTTG	135 bp	HP200561
	R	ATGGGCTACAGGCTTGTCACTC		
NOS2	F	GCTCTACACCTCCAATGTGACC	136 bp	HP200591
	R	CTGCCGAGATTTGAGCCTCATG		
GAPDH	F	GTCTCCTCTGACTTCAACAGCG	131 bp	HP205798
	R	ACCACCCTGTTGCTGTAGCCAA		
